# Understanding the determinants of under-five child mortality in Uganda including the estimation of unobserved household and community effects using both frequentist and Bayesian survival analysis approaches

**DOI:** 10.1186/s12889-015-2332-y

**Published:** 2015-10-01

**Authors:** Justine B. Nasejje, Henry G. Mwambi, Thomas N. O. Achia

**Affiliations:** School of Mathematics, Statistics and Computer Science, University of KwaZulu-Natal, 22 St. Patricks road, Scottsville, Pietermaritzburg South Africa; Division of Epidemiology and Biostatistics, School of Public Health, University of Witwatersrand, Witwatersrand, South Africa

## Abstract

**Background:**

Infant and child mortality rates are among the health indicators of importance in a given community or country. It is the fourth millennium development goal that by 2015, all the United Nations member countries are expected to have reduced their infant and child mortality rates by two-thirds.

Uganda is one of those countries in Sub-Saharan Africa with high infant and child mortality rates, therefore it is important to use sound statistical methods to determine which factors are strongly associated with child mortality which in turn will help inform the design of intervention strategies

**Methods:**

The Uganda Demographic Health Survey (UDHS) funded by USAID, UNFPA, UNICEF, Irish Aid and the United Kingdom government provides a data set which is rich in information on child mortality or survival. Survival analysis techniques are among the well-developed methods in Statistics for analysing time to event data. These methods were adopted in this paper to examine factors affecting under-five child mortality rates (UMR) in Uganda using the UDHS data for 2011 in R and STATA software.

**Results:**

Results obtained by fitting the Cox-proportional hazard model with frailty effects and drawing inference using both the frequentists and Bayesian approaches at 5 % significance level, show evidence of the existence of unobserved heterogeneity at the household level but there was not enough evidence to conclude the existence of unobserved heterogeneity at the community level. Sex of the household head, sex of the child and number of births in the past one year were found to be significant. The results further suggest that over the period of 1990–2015, Uganda reduced its UMR by 52 % .

**Conclusion:**

Uganda has not achieved the MDG4 target but the 52 % reduction in the UMR is a move in the positive direction. Demographic factors (sex of the household head) and Biological determinants (sex of the child and number of births in the past one year) are strongly associated with high UMR. Heterogeneity or unobserved covariates were found to be significant at the household but insignificant at the community level.

## Background

Infant and child mortality rates are important indicators of societal and national development as they serve as key markers of health equity and access [1, 2]. Despite huge investments by national governments and developmental partners in improving access to health care, the reduction of infant and child mortality rates by two-thirds between 1990–2015 as stipulated in the fourth Millennium Development Goal (MDG4) [3, 4] has not been attainable within low and middle income countries [5].

In response to the MDG4 most countries in the Sub-Saharan Africa region have instituted mechanisms and policies aimed at addressing weaknesses in their health systems and engaging policy makers to look at inequalities in outcomes. Despite these measures, most countries in the region have not met the MDG4 target [6]. Uganda in East Africa is a low income Sub-Saharan African country with a high UMR. The UMR for the five years immediately preceding the 2011 Uganda Demographic and Health survey (corresponding roughly to 2006–2010) was reported to be 90 deaths per 1,000 live births [7]. Previous studies have not suggested declines in UMR in Uganda. Over the period 1995–2000, the UMR increased from 147.3 to 151.5 deaths per 1000 live births. There is further evidence in literature that the UMR remained unchanged in the period of 1991–1995 but declined in the period of 2001–2005 to 125 deaths per live births. These disconcerting figures can be attributed to numerous factors, key among them is the heavy HIV/AIDS burden in Uganda. In 2011 there were an estimated 1.4 million people living with HIV/AIDS in Uganda, of whom an estimated 190,000 were children. An estimated 62,000 people died from AIDS in 2011 and 1.1 million children have been orphaned by the devastating epidemic in Uganda [8].

In order to develop measures to reduce infant and child mortality rates, an assessment of individual and contextual determinants of child survival is necessary [4]. Based on existing theoretical frameworks, sex of the child, place of residence, birth intervals and maternal education have been identified as significant predictors of child survival. Within the Ugandan context, previous studies suggest a need to identify determinants of child survival so as to design relevant interventions and programs, appropriate to local and national needs [9]. Most studies on child survival in Uganda have employed standard survival methodologies, like the Cox-proportional hazard model, to identify factors associated with child mortality, ignoring unobserved heterogeneity at cluster or household level. This study uses a shared frailty model within the Bayesian Integrated Nested Laplace Approximation (INLA) paradigm [10, 11] to investigate determinants of UMR in Uganda.

## Methods

### The data

The data used in this study was collected during the 2011 Uganda Demographic Health Survey (UDHS) which was carried out from May through December 2011 [12]. This was the fifth comprehensive survey conducted in Uganda as part of the world wide demographic and health surveys [13].

A representative sample of 10,086 households was selected during the 2011 UDHS. The sample was selected in two stages. A total of 404 enumeration areas (EAs) were selected from among a list of clusters sampled for the 2009/10 Uganda National Household Survey (2010 UNHS). In the second stage of sampling, households in each cluster were selected from a complete listing of households. Eligible women for the interview were aged between 15–49 years of age who were either usual residents or visitors present in the selected household on the night before the survey. Out of 9,247 eligible women, 8,674 were successively interviewed with a response rate of 94 % (91 % in urban and 95 % in rural areas). The study population for this analysis includes infants born between exactly one and five years preceding the 2011 UDHS; who were the outcomes of singleton deliveries and who either survived the infancy period or not.

Children born to women aged between 15–49 years of age from 4285 households and 404 communities were considered for this analysis. One has to note that we excluded children that died before one month (28 days and below) and it is also important to note that we excluded all births in the year 2011 (the year of the survey). The number of observation at this level was 6,692 representing the number of children dead or alive, born in the period of five years preceding the date of the survey.

### The outcome variable

*Under-five child mortality is defined as mortality from the age of 1 months to the age of 59 months. Therefore, the dependent variable in this study is* “*the risk of death occurring in an age interval in the 1–59 month period”. The outcome variable was thus survival time in months of the children under the age of five.*

### Explanatory variables

Based on a literature survey [4, 14, 15] and limitations like high level of missingness in the dataset used, we assessed the nature of the response variables and the following covariates: *mother’s age group (less than 20 years, 20–29,30-39,40 + years); type of residence (Urban, Rural); mother’s level of education (illiterate, primary, secondary and higher); partner’s level of education (Illiterate, primary, secondary and higher); birth status (Singleton birth, multiple births); sex of the child (male, female); wealth index (poorest, poorer, middle, richer, richest); children ever born (one child, two children, three children, four and more); birth order (first child, second to third child, 4th-6th child); religion (Catholic, Muslim ,other Christians, others); types of toilet facility (flush toilet, pit latrine, no facility); mother’s occupation (not-working, sales and service, agriculture); births in the past one year (no births, one birth, two births); children under the age of five in the household (no child, one child, two children, three, four); sex of the household head (male, female); source of drinking water (piped water, borehole, well, surface/rain/pond/lake, others); mother’s age at first birth (less than 20 years, 20–29, 30–39 years).*

### Preliminary survival analysis

The Schoenfeld residual test [16] was carried out in the R software using the **cox.zph** command. Under this approach it is assumed that the regression parameters of covariates do not vary with time. All those whose regression parameters changed with time do not satisfy the proportional hazard assumption and were therefore not included in the final Cox-PH model. The results of this analysis have been presented in Table 2.

The estimation and results were performed using the R software [17].

Three non-Bayesian models were considered. The first model (Model I) was the standard Cox Proportional Hazards model; the second (Model II) was a model with a household specific frailty term; and the final model (Model III) was a model with a community specific frailty term. Appendix 1 provides a detailed mathematical description of these models.

### Bayesian survival analysis

In this study, a model that assumed that the time to death of the children under-five followed a Weibull distribution was used. The Weibull model for time to event is a popular parametric model because it inherently relaxes the assumption of constant hazard as is the case with the exponential distribution. This model was implemented with and without family and community effects so as to investigate the main factors that affect UMR in Uganda. Bayesian inference was carried out using the R library INLA [18] which implements the Integrated Nested Laplace approximation approach for latent Gaussian models [11].

Four distinct Bayesian survival models were considered: the first (Model IV) was a Bayesian Weibull survival model; the second (Model V) was a Bayesian Cox-PH model; the third (Model VI) was Bayesian (Weibull) model with community level frailty; and finally (Model VII) was a Bayesian (Weibull) model with household level frailty. Appendix 2 provides a detailed mathematical description of these models.

### Analysis approach

Data analysis was done by using R and STATA software. The R libraries Mass, survival and packages *frailty pack* were used for the analysis of the data. STATA inbuilt commands for survival analysis were used to do the analysis under the frequentist approach. STATA command *stptime* was used to compute mortality rates. The main reason for using both the frequestist and Bayesian approaches was to have confidence in the results when the two approaches agree.

## Results

Five years prior to the survey, Uganda had an UMR of 90 per 1000 live births which was almost 15 times the average rate in high-income countries (6 deaths per 1000 live births) [19]. The MDG4 target is aimed at reducing UMR by two-thirds. The time this target was set Uganda had an UMR of 147 per 1000 live births. From the analysis, the UMR is estimated to be 71.28 per 1000 live births, which is still high compared to the global UMR of 46 per 100 live births [19]. Despite the fact that Uganda has not achieved the MDG4, the results from our analysis suggest that the UMR for the country has reduced by 52 %.

Table 1 shows the distribution of deaths of the children under the age of five at each factor level included in the analysis.

**Table 1:** Distribution of births and deaths by survival determinants

Variable	N (%)	Variable	N (%)
*Mother’s education level*		*Children ever born*	
Illiterate Mothers	4493 (7.7)	One child	601 (3.3)
Mother completed primary	1868 (6.4)	Two children	1146 (7.1)
Secondary and higher	331 (4.2)	Three children	1020 (6.6)
*Partner’s level of education*		Four and more	3925 (7.9)
Illiterate Father	3446 (7.7)	*Birth order number*	
Father completed primary	2457 (6.9)	First child	1249 (7.6)
Secondary and higher	789 (5.2)	Second to Third child	2091 (5.6)
*Birth status*		4th-6th child	2098 (7.1)
Singleton births	6479 (6.7)	7-th + child	1254 (9.2)
Multiple births (Twins)	213 (21.5)	*Religion*	
*Sex of the child*		Catholics	2939 (7.4)
Males	3325 (7.8)	Muslims	921 (7.5)
Females	3367 (6.3)	Other Christians	2758 (6.8)
*Type of place of residence*		Others	74 (5.4)
Urban	1389 (5.8)	*Type of toilet facility*	
Rural	5303 (7.5)	Flush toilet	121 (4.1)
*Wealth index*		Pit latrine	5407 (6.9)
Poorest	1754 (7.5)	No-facility	1164 (8.2)
Poorer	1317 (8.5)		
Middle	1195 (7.2)		
Richer	1041 (6.9)		
Richest	1385 (5.5)		

In this study most of the variables considered were categorical. For variables which were not categorical, their categorizations were adopted from previous research [20–24].

Table 1 presents the distribution of death of children under the age of five for each covariate considered in the analysis. It shows that among the illiterate mothers, out of the 4493 children born, 7.7 % died before celebrating their fifth birthday which was the highest death proportion followed by mothers who had completed primary education with 6.7 % of the deaths and lastly, mothers who had acquired secondary and higher education with 4.2 % proportion of deaths. The table also summarises the distribution of deaths and births of children for all the other covariates considered in this study.

Table 2 presents the results for testing the proportional hazard assumption. Mother’s education, total number of children ever born, type of place of residence, type of birth, previous birth interval and wealth index violated the proportionality hazard assumption(p-values less than 0.05). They were therefore not included in the fitted Cox-PH model. The other variables like sex of the household head, father’s education, sex of the child, and number of births in the past one year, mother’s age group and mother’s age at first birth are the only factors that satisfied the PH assumption and were therefore included in the final model.

**Table 2:** Testing the proportional hazard assumption

Variable	Chi-square	*p*-value	Variable	Chi-square	*p*-value
Mother’s education			Number of births in the past one year		
No formal	1.00 (Ref)		No birth	1	
Primary	4.83	0.03	1 birth	0.7	0.4
Secondary and higher	7.52	(<0.01)	2 births	1.24	0.27
GLOBAL	11.25	(<0.01)	GLOBAL	1.81	0.4
Father’s education			Number of births in the last five years		
No formal	1.00 (Ref)		1 births	1	
Primary	0.51	0.48	2 births	0.11	0.75
Secondary and higher	0.86	0.35	3 births	0.03	0.86
GLOBAL	1.12	0.57	4+ births	5	0.03
Sex of the child			GLOBAL	5.85	0.12
Male	1.00 (Ref)		Mother’s age		
Female	1.99	0.16	Less than 20 years	1.00 (Ref)	
Total number of children ever born			20-29 years	0.16	0.69
1 child	1.00 (Ref)		30-39 years	0.63	0.43
2	5.39	0.02	40+ years	0.08	0.78
3	0.44	0.51	GLOBAL	5.58	0.13
4+	0.26	0.61	Sex of household head		
GLOBAL	14.61	(<0.01)	Male	1.00 (Ref)	
Type of place of residence			Female	0.07	0.79
Rural	1.00 (Ref)		Source of drinking water		
Urban	8.43	(<0.01)	Piped water	1.00 (Ref)	
Wealth index			Borehole	0.17	0.68
Poorest	1.00 (Ref)		Well water	0.12	0.73
Poorer	0.17	0.7	Surface/pond/lake/Rain/etc.	2.58	0.11
			Others	1.82	0.18
Middle	0	0.98	GLOBAL	6.55	0.16
Richer	6.94	(<0.01)	Mother’s occupation		
Richest	2.26	0.13	Not working	1.00 (Ref)	
GLOBAL	9.29	0.05	Sales and Services	0.202	0.65
Birth order			Agriculture	6.88	(<0.01)
1St	1.00 (Ref)		GLOBAL	14.41	(<0.01)
2nd	0.28	0.59	Type of birth		
3rd	6.69	(<0.01)	single birth	1.00 (Ref)	
4-th	2.64	0.1	Multiple births	13	(<0.01)
GLOBAL	8.46	0.04	Religion		
Age at first birth			Catholic	1.00 (Ref)	
<20 years	1.00 (Ref)		Muslim	0.009	0.92
20−29 years	0.1	0.75	Other Christians	0.73	0.39
30+ years	0.41	0.52	Others	1.59	0.21
GLOBAL	0.54	0.76	GLOBAL	2.21	0.53
Previous birth interval					
<2 years	1.00 (Ref)				
2 years	1.83	0.18			
3 years	0.97	0.32			
4 years	2.53	0.11			
GLOBAL	8.69	0.03			

Table 3 presents the factors that were strongly associated with high UMR. This table also summarizes the results of all the models considered for the frequentist approach.

**Table 3:** Best fitting model for the Standard Cox proportional hazard model

		Model I	Model II	Model III
Variable	HR (95 % CI)	AHR (95 % CI)	AHR (95 % CI)	AHR (95 % CI)
Sex of the child				
Male		1.00	1.00	1.00
Female		0.83* (0.69, 0.99)	0.83 (0.68,1.00)	0.84 (0.70,1.00)
Father’s education				
No formal		1.00	1.00	1.00
Primary		0.90 (0.75 ,1.09)	0.95 (0.77,1.18)	0.95 (0.78,1.16)
Secondary and Higher		0.66* (0.47,0.92)	0.73(0.50,1.06)	0.74 (0.53,1.05)
Age at first birth				
Less than 20 years			1.00	1.00
20-29			0.84 (0.67,1.07)	0.86 (0.70,1.06)
30-39			1.89 (0.49,7.32)	1.66 (0.52,5.28)
Number of births in the past one year				
No birth		1.00	1.00-	1.00-
One birth		1.22* (1.01,1.48)	1.25* (1.01,1.55)	1.25* (1.03,1.52)
Two births		2.51* (1.04, 6.09)	4.57* (1.36,15.32)	2.76* (1.11,6.85)
Sex of household head				
Male		1.00	1.00	1.00
Female		1.33* ( 1.09,1.62)	1.39* (1.11,1.74)	1.36* (1.11,1.66)
Mother’s age group				
Below 20 years		1.00	1.00	1.00
20-29 years		0.68 (0.46 ,1.01 )	0.89 (0.59,1.33)	0.84 (0.57,1.22)
30-39 years		0.77 (0.51,1.14 )	1.00 (0.65,1.52)	0.94 (0.63,1.39)
40+ years		0.95 ( 0.59,1.51 )	1.28 (0.77,2.12)	1.18 (0.74,1.88)
Source of drinking water				
Piped water			1.00	1.00
Borehole			1.23 (0.92,1.65)	1.21 (0.92,1.61)
Well			1.16 (0.82,1.62)	1.15 (0.84,1.59)
Surface/Rain/Lake			1.38 (0.95,2.00)	1.36 (0.96,1.92)
Others			1.34 (0.79,2.28)	1.30 (0.80,2.11)
Religion				
Catholic			1.00	1.00
Muslim			1.05 (0.78,1.43)	1.05 (0.79,1.39)
Other Christians			0.95 (0.76,1.18)	0.96 (0.79,1.18)
Others			0.64 (0.22,1.86)	0.69 (0.25,1.89)
Household Frailty parameter (Variance)			=1.78 (0.48)	
Community Frailty parameter (Variance)				=0.12 (0.07)
Penalised Marginal loglikelihood			−3025.98	−3042.18

Based on the Cox-PH model, the number of births in the past one year and the sex of the household head were found to be strongly associated with high mortality rates. The children whose mothers had more than one birth in the past on year were at a higher risk of death than those whose mothers had no birth at all. The children born in households headed by women were at a high risk of death than those born in households where the man is the head. A study done by [25, 26], pointed out factors associated to UMR in Uganda as; mother’s education, sex of the child, place of residence, birth intervals, household size, mother’s age at first birth, duration of breast feeding, household hardship, place of delivery and mother’s education. Some of which agree with the results from our study.

**Fig. 1:**
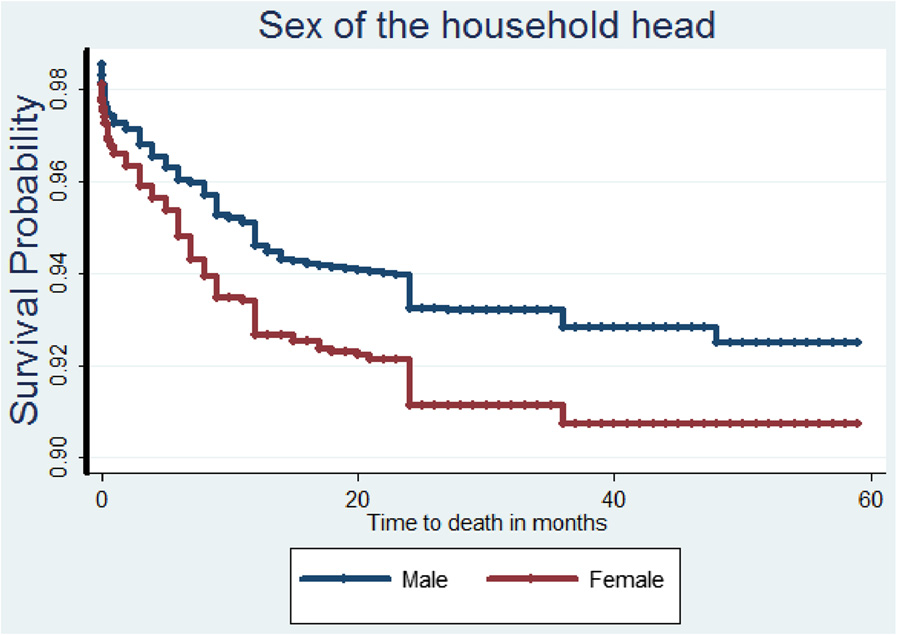
The estimated survival curve for children under the age of five in female headed households is above that of male headed households. This implies that female headed households are associated to a low under-five child survival rate

**Fig. 2:**
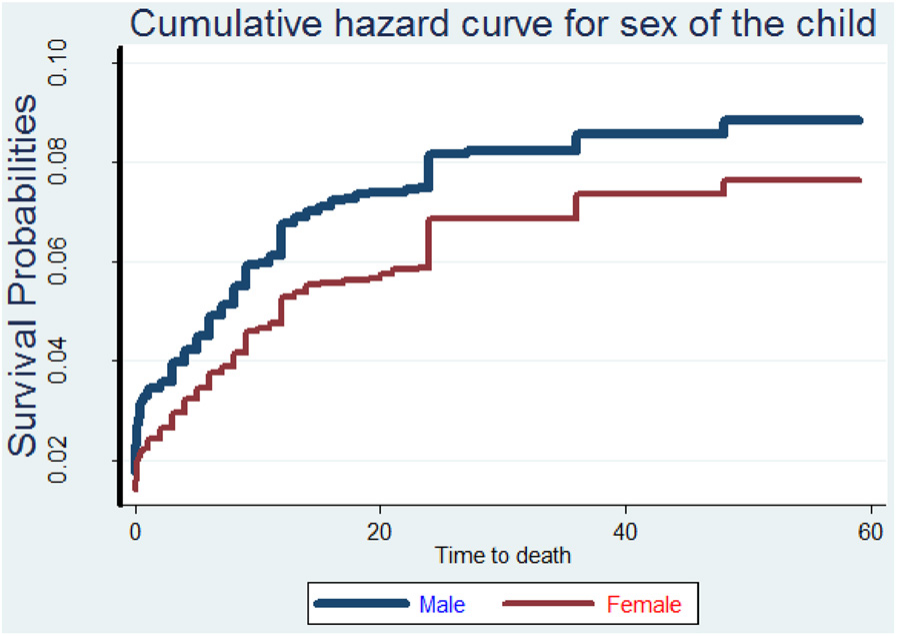
The estimated cumulative hazard curve for the male children is above that of the female children indicating that boys are at a higher risk of death before celebrating their fifth birthday than girls .

**Fig. 3:**
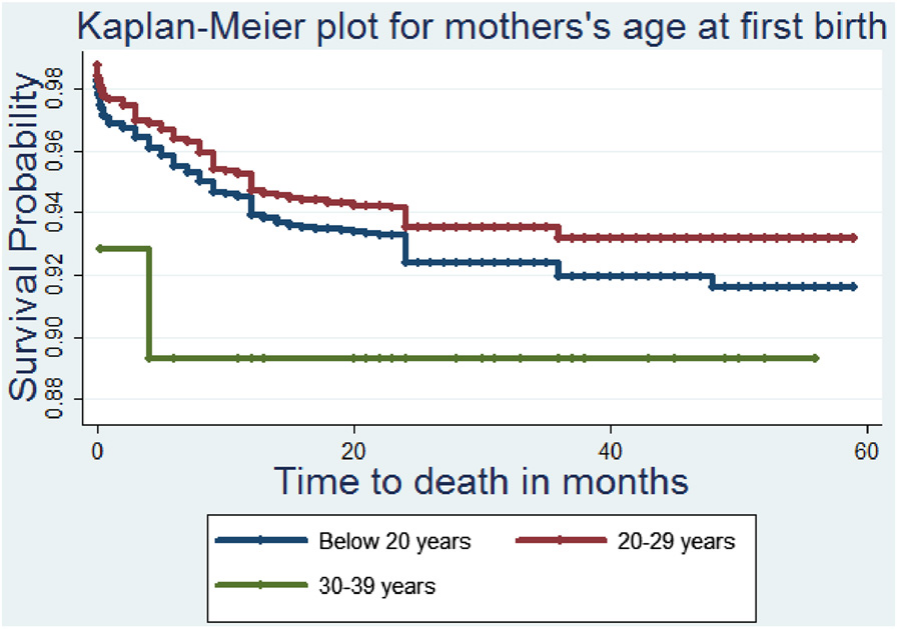
The estimated survival curves show that women whose age at first birth was below 20 years and that of those who were above 30 years put their children at a high risk of death before celebrating their fifth birthday

**Fig. 4:**
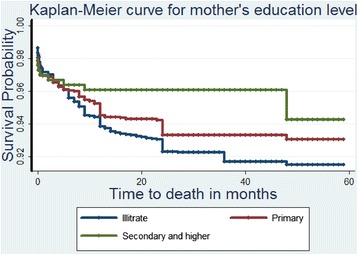
The estimated survival curves show that women with secondary school and higher education increased the chance of survival for their children under the age of five. The women with no formal education put their children below the age of five at a higher risk of death

Lastly, Table 4 presents the results from the Bayesian analysis which leads to generally similar results but identifies another factor strongly associated to a high UMR as mothers’ age group. Figs. 1, 2, 3, and 4 show the survival and the cumulative hazard curves for selected covariates considered in this study. These figures confirm the results presented in Tables 1, 2, 3, and 4 for these covariates.

**Table 4:** Parameter estimates, 95 % Credible Intervals for Bayesian models considered

	Model IV: Weibull model	Model V: Bayesian Cox-PH model	Model VI: Bayesian (Weibull) model with community level frailty	Model VII: Bayesian (Weibull) model with household level frailty
Factors	Mean	Mean	Mean	Mean
Intercept	−3.52 (−3.99,-3.08)	−5.67 (−6.13,-5.23)	−3.49 (−3.97,-3.05)	−3.89 (−4.42,-3.39)
Fixed effects				
Father’s education				
Illiterate	Ref	Ref	Ref	Ref
Complete Primary	−0.08 (−0.28, 0.11)	−0.09 (−0.28, 0.11)	−0.08 (−0.28,0.11)	−0.08 (−0.28,0.12)
Secondary and higher	−0.33 (−0.69, 0.00)	−0.34 (−0.69, 0.01)	−0.33 (−0.69,0.00)	−0.34 (−0.70,0.01)
Sex of the child				
Male	Ref	Ref	Ref	Ref
Female	−0.19* (−0.37, −0.01)	−0.19* (−0.37, −0.01)	−0.19* (−0.37,-0.01)	−0.19* (−0.38,-0.01)
Age at first birth				
Less than 20 years	Ref	Ref	Ref	Ref
20−29 years	−0.14 (−0.36, 0.07)	−0.11 (−0.33, 0.09)	−0.14 (−0.36,0.06)	−0.16 (−0.38,0.06)
30−39 years	0.49 (−0.78,1.52)	0.59 (−0.69, 1.62)	0.49 (−0.78,1.52)	0.56 (−0.78,1.67)
Births in the past one year				
No-births	Ref	Ref	Ref	Ref
One birth	0.19 (0.00,0.39)	0.25 (0.05, 0.44)	0.19 (0.00,0.39)	0.19 (−0.01,0.39)
Two births	0.98* (0.02,1.79)	1.19* (0.22, 1.99)	0.99* (0.12,1.79)	1.19* (0.12,2.10)
Sex of the household head				
Male	Ref	Ref	Ref	Ref
Female	0.29* (0.09,0.49)	0.29* (0.09, 0.49)	0.29* (0.09,0.49)	0.30* (0.09,0.51)
Mother’s age group				
Below 20 years	Ref	Ref	Ref	Ref
20−29 years	−0.37 (−0.75,0.03)	−0.61* (−0.98, −0.19)	−0.37 (−0.75,0.04)	−0.35 (−0.75,0.08)
30−39 years	−0.27 (−0.66,0.15)	−0.53* (−0.92, −0.11)	−0.26 (−0.65,0.15)	−0.24 (−0.65,0.19)
40+ years	−0.06 (−0.52,0.42)	−0.37 (−0.84, 0.10)	−0.05 (−0.52,0.43)	−0.02* (−0.51,-0.49)
Source of drinking water				
Piped water	Ref	Ref	Ref	Ref
Borehole	0.12 (−0.15,0.39)	0.12 (−0.15, 0.39)	0.12 (−0.15,0.39)	0.12 (−0.15,0.41)
Well	0.06 (−0.25,0.37)	0.06 (−0.25, 0.37)	0.06 (−0.25,0.37)	0.06 (−0.26,0.39)
Surface/Rain/Pond/Lake/Tank	0.24 (−0.09,0.58)	0.24 (−0.09, 0.58)	0.24 (−0.10,0.58)	0.24 (−0.11,0.59)
Others	0.18 (−0.31,0.65)	0.17 (−0.33, 0.63)	0.18 (−0.31,0.65)	0.20 (−0.31,0.69)
Precision for baseline Hazard		18409.77 (1271.41, 67216.46)		
Random effects				
Precision for frailty term			64.88 (11.66,63.47)	0.35 (0.91,2.28)
Alpha parameter for Weibull	0.33 (0.30,0.36)		0.19 (1.07,1.29)	0.01 (0.30,0.36)
Marginal Likelihood		−3312.13	−2951.26	−2945.52

By using the likelihood ratio test with a null hypothesis that the variance of the community frailty term is zero (*θ* = 0), the chi-square test statistic yielded a *p*-value of 0.052. At 0.05 level of significance, it implies that there is not enough evidence to show the existence of unobserved heterogeneity at community level. This statement implies that the survival times of children under the age of five within the same community can be well explained by the observed covariates considered in the study using the hazard ratios presented by the results from the analysis without the community frailty term. In this case therefore one can use the standard Cox-PH model because the results suggest that there is no difference on the conclusions that would be drawn about the data set.

In the case of household or family frailty, there were 4285 households in the sample considered for analysis. The variance component of the frailty term (household frailty) is *θ* = 1.78 , which is significantly different from zero, and gives evidence of the existence of the unobserved heterogeneity at family or household level. This implies that there are other factors affecting under-five child mortality at household level that are not explained by the observed covariates included in the model. The sources of the unobserved heterogeneity at the household level can be attributed to access to food, child care, sanitation and other factors that cannot be easily measured or observed at household level. Note that the variables which failed the PH assumption and were not modelled could contribute to the significance of this effect. The results further suggest that some households were associated with a higher risk of children dying before celebrating their fifth birthday than others. However this is an area which needs further research in order to explain the reasons for this unobserved heterogeneity at a local level.

These results indicate that more efficient interventions may be those that target individual households rather than communities. Interventions among many may include visits by the health or government officials to the households with children under the age of five to record any vital information on their health and household environment, educating women on health care for children under the age of five at household level and lastly door to door education for women at child bearing age on contraception . These interventions may be expensive but may realistically help reduce the high UMR in Uganda and hence help in achieving or realising the millennium development goal.

## Discussion

The UMR estimated in this paper indicates a decline in the UMR for Uganda but still high compared to target rate. This suggests that the MDG4 target for Uganda has not been met despite the fact that we have reached the deadline. Uganda just like the other sub-Saharan Africa countries has not met the MDG4 target but is showing a steady decline in national UMR.

Number of births in the past one year, sex of the child and sex of the household head are the factors associated with increased risks of UMR in Uganda. All the above mentioned factors relate to inappropriate child spacing, customs and norms practised by families and communities, and lastly family related problems (Family violence). Similar results have been reported elsewhere in the literature [14, 20, 27]. From the results, women who had given birth to more than two children in the year had their children at a higher risk of death before reaching the age of five. This factor explains the inappropriate child birth intervals and may be a result of lack of knowledge of the available family planning methods. As evidenced from the data, only 25 % of the women in the sample were using Modern family planning methods like injections, pills among others, 3.4 % were using traditional methods, 47 % were non-users and intend to use later and lastly 23 % of these women did not intend to use these family planning methods at the time of the survey. The health facilities where these women go for antenatal care have failed to inform the women about the family planning methods available to them , 50 % of the women confessed that the health facilities did not inform them about family planning and only 27 % of the women claimed to have been well informed and 22 % of these women had missing information. This article supports the view that mothers or women should be made more aware of the contraception options available to increase birth intervals. This would lead to a reduction in UMR in the country and hence help the country to achieve the MDG4 sometime in the future.

Male children were at a high risk of death than their female counterparts. This may be due to the fact that majority of the tribes in the country have a cultural norm of viewing the girl child as a source of wealth through bride price [28]. In order to achieve the MDG4 target, policies that target factors like education, poverty reduction among others especially with emphasis in rural communities will help to break such cultural norms.

Female headed households were associated with an increased risk of UMR than those that are headed by the males. Since in most of the tribes in the country, a man is considered to be the bread winner and head of the household, finding a household headed by a woman is directly linked to a family that is insecure in a number of ways such as food availability and previous history of home violence. These women choose to leave their original marital homes with their children because of such ills. This is a problem because most of the women cannot work and at the same time take care of the family which are often large. Laws on marriage aimed at protecting women and children from domestic violence and also addressing the issue of who takes care of the children in case of a divorce should be passed by the legislature. These laws need to be enforced at the local administrative level rather than only being discussed at the national level. Education for the girl child should be emphasized so that women are fundamentally and financially capable of taking care of their children in the event of a separation. As evidenced from the data, the types of jobs most of these women do are odd jobs due to their low level of education. The data suggests that 789 men had acquired secondary and higher level of education and less than half that number for women had aquired the same level of education (331 women had secondary and higher education) .

The household and community variations summarise the effects of biological, parental competence, genetic, customs and other unobserved factors that are not accounted for by the fixed effects at household and community level respectively.

The results suggest that deaths tend to cluster in some households and to a smaller extent in some communities.

## Conclusions

The UMR of 71.28 [95 % CI:65.11-77.44] per 1000 live births indicates a decline in the UMR for Uganda but still lagging behind on the achievement of the MDG4 target despite the deadline. Government interventions must address issues like passing the marriage law to ensure that children under the age of five are safe even after the divorce of their parents. Education of a girl child especially in the rural communities should be emphasized to break the cultural norms like taking the child (girl child) as a family wealth through bride price.

The results also suggest that government interventions should focus on small communities containing few household rather than a big community in order to reduce on the heterogeneity across households.

The paper also shows that the results from the Bayesian approach are consistent with those from the frequetist approach but in most of the research papers on under-five mortality, researchers have ignored the use of the Bayesian approaches despite their advantages over the Frequestists approaches.

### Limitations of the analysis

Demographic health survey datasets are cross-sectional in nature and therefore prone to problems like high level of missingness due to failure of the respondents in recalling past events and the fact that some covariates which could help in the analysis may not be captured in the survey. The high level of missingness was evident in the 2011 UDHS dataset and among the covariates that had a high level of missingness include; birth intervals both preceding and succeeding with 1261 and 3812 cases respectively and number of antenatal visits with 2950 missing cases. Thus possible extensions for further research include the use of models that account for missing data in surveys as well as considering more flexible survival analysis models that do not necessarily rely on the proportional hazards assumption. More advanced methods like survival trees and random survival forests are also better options when analyzing large datasets and identifying more frail groups (frailty effects).

### Strengths of the analysis

We used Bayesian inference. This is very special because Bayesian approaches have been found to have some advantages over the frequestist approaches. Below are some of the advantages of using a Bayesian approach for analyzing data over the frequentist approach:

1. Bayesian models allow for informative priors such that prior knowledge can be used to inform the current model.
2. Bayesian inference assumes (the data to be fixed) the observed data is fixed and the unknown parameters to be random which is the opposite of the frequentist inference. The Frequentists estimation is therefore not based on the data at hand but data at hand plus hypothetical repeated sampling in future with similar data.
3. There is no Frequentists probability distribution associated with the unknown parameters or hypotheses. Bayesian inference therefore estimates a full probability model.
4. Bayesian inference estimates the probability of the hypothesis given the data were as the frequentists estimate the probability of the data given the hypothesis. Hypothesis testing itself suggests that one should test for the hypothesis given the data.

Other strengths of the analysis are derived from the fact that we have used the Integrated Nested Laplace Approximation(INLA) for Bayesian inference. This is a simple but powerful tool for Bayesian inference. The other tools for Bayesian inference have not been programmed to handle large data sets as this (over 6000 cases) and in case one succeeds with programming it for a large dataset, it takes days or even weeks to get the results. With INLA the model that took the longest time took about 1081.709 seconds to run and the results are known to be close to those one would get if they used other software’s for Bayesian like WinBUGS[18].

## Appendix 1

Survival analysis techniques were used in this paper on the 2011 Uganda Demographic Health Survey data to examine the effect of frailty and other factors provided in the data set on under-five children survival in Uganda.

The Cox-proportional hazard model is a more general and the most commonly used model in modelling the hazard and survival function. The Cox model [29] has the from:

Model **1**: *h*(*t*|**X**_*i*_) = *h*_0_(*t*)exp(**X**_*i*_^*T*^**β**).

The Cox-proportional hazard model assumes a proportional hazard implying that the model cannot be used in situations where the assumption is violated. Its strength lies in its ability to leave the baseline hazard unspecified and not dependent on any parametric distribution which may not be easy to discern.

Let *N* denote the number of children considered in the UDHS 2011 data set with each child in the data set belonging to a given household found in a given community. Let the total number of households or communities be denoted by *G* such that, given the *i-*th household or community that consists of *n*_*i*_ children under the age of five, then;

$$ {\displaystyle \sum_{i=1}^G{n}_i}=N. $$

We define the variable

$$ \delta =\left\{\begin{array}{l}1,\kern1em \mathrm{child}\ \mathrm{is}\ \mathrm{dead}\ \mathrm{at}\ \mathrm{the}\ \mathrm{time}\ \mathrm{of}\ \mathrm{interview}\\ {}0,\kern1em \mathrm{child}\ \mathrm{is}\ \mathrm{still}\ \mathrm{alive}\ \mathrm{at}\ \mathrm{the}\ \mathrm{time}\ \mathrm{of}\ \mathrm{interview}.\end{array}\right. $$

as the censoring indicator. The hazard function of the *j*-th child of the *i*-th household or community is given as:

Model **2**: *h*_*ij*_ = *h*_0_(*t*) exp (**X**_*ij*_^*T*^ + *u*_*i*_)

where **X**_*ij*_ is a vector of covariates for child *j* in the *i*-th household or community, *u*_*i*_ the unobserved covariates and *h*_0_(*t*) denotes the baseline hazard function.

As per the above model formulation it implies that the variable *z*_*i*_ = exp (*u*_*i*_) is the frailty term. The hazard function can therefore be written as:

Model 3: *h*_*ij*_(*t*) = *h*_0_(*t*)exp(**X**_*ij*_^*T*^**β** + *u*_*i*_) = *z*_*i*_*h*_0_(*t*)exp(**X**_*ij*_^*T*^**β**)

Note that it is assumed that the *Z*_*i*_’s are independent with an identical probability density function denoted as *f*(*z*).

## Appendix 2

### The Weibull model

Let *t*_*i*_ denote the survival time of the *i* − th child. We can assume that *t*_*i*_ has a Weibull distribution with parameters *α* > 0 and*λ*, with a density function of the form:

$$ f\left({t}_i\Big|\alpha, \lambda \right)=\alpha {t}_i^{\alpha -1} \exp -{\left[\lambda {t}_i\right]}^{\alpha },\kern1em 0<{t}_i<\infty . $$

The survival function of *t*_*i*_ is given by *S*(*t*_*i*_|*α*, *λ*) = exp ( − exp (*λ*)*t*_*i*_^*α*^) .

The likelihood function of the unknown parameters (*α*, *λ*) given the data can be written as:

$$ \begin{array}{c}L\left(\alpha, \lambda \Big|D\right)={\displaystyle \prod_{i=1}^nf}\left({t}_i\Big|\alpha, \lambda \right)S{\left({t}_i\Big|\alpha, \lambda \right)}^{\left(1-{\delta}_i\right)}\\ {}={\alpha}^{{\displaystyle \sum {\delta}_i}} \exp \left\{\lambda {\displaystyle \sum_{i=1}^n{\delta}_i}+{\displaystyle \sum_{i=1}^n\left({\delta}_i\left(\alpha -1\right) \log \left({t}_i\right)- \exp \left(\lambda \right){t}_i^{\alpha}\right)}\right\}\end{array} $$

where *δ*_*i*_ is an indicator variable taking value 1 if *t*_*i*_ is the failure time and 0 if *t*_*i*_ is right censored.

To incorporate covariates we therefore write*λ* = *X*_*i*_^’^*β*, where *X*_*i*_ and *β* are *p* × 1 vector of covariates and regression coefficients respectively.

Assuming gamma prior with parameters (*α*_0_, *κ*_0_) for *α* and normal prior with parameters (*μ*_0_, *σ*_0_^2^) for *λ*, the joint posterior distribution of (*α*, *λ*) is given by

$$ \pi \left(\alpha, \lambda \Big|D\right)\propto L\left(\alpha, \lambda \Big|D\right)\pi \left({\alpha}_0{\kappa}_0\right)\pi \left(\lambda \Big|{\mu}_0,{\sigma}_0\right) $$

If we assume a normal prior *N*_*p*_ (*μ*_0_, ∑ _0_) for *β*, the joint posterior is given by

$$ \pi \left(\beta, \alpha \Big|D\right)\propto {\alpha}^{\alpha_0+d+1} \exp {\displaystyle \sum_{i=1}^n\left({\delta}_i+{\boldsymbol{X}}_i^{\hbox{'}}\beta +{\delta}_i\left(\alpha -1\right) \log \left({t}_i\right)\right)-{t}_i^{\alpha } \exp \left({\boldsymbol{X}}_i^{\hbox{'}}\beta \right)-{\kappa}_0\alpha -\frac{1}{2}\left(\beta -{\mu}_0\right){\varSigma}_0^{-1}\left(\beta -{\mu}_0\right)} $$

where *D* = (*n*, *t*, *X*, *δ*) denote the observed data for regression model and *X* is the *n* × *p* matrix of covariates with the *i* − th row as *X*_*i*_ and lastly *δ* = (*δ*_1_, …, *δ*_*n*_)’.

### The Weibull frailty model

Let *t*_*ij*_ be the survival time for the *j* − th child in the *i* − th household (or cluster), *i* = 1, …, *n* and *j* = 1, …*m*_*i*_. Here *m*_*i*_ represent the number of individual in the *i* − th cluster. We assumed that these *t*_*ij*_ follow i.i.d. Weibull distribution such that

$$ {t}_{ij}\sim Weibull\left(\alpha, {\eta}_{ij}\right),\kern1em \alpha \kern0.5em >0, $$

For frailty models the conditional hazard function of *t*_*ij*_ given the unobserved frailty*z*_*i*_, a covariate vector *X*_*ij*_ and the Weibull parameter *α* is given by;

$$ h\left({t}_{ij}\Big|{X}_{ij},{z}_i,\alpha \right)=\alpha {t}_{ij}^{\alpha -1} \exp \left[{\eta}_{ij}\right], $$

where *η*_*ij*_ = *β*_0_ + *X*_*ij*_^*T*^*β* + *z*_*i*_, *β* is a *p* × 1 vector of regression coefficients and *β*_0_ denotes the intercept and *X*_*ij*_ is a *p* × 1 covariate vector. The complete data likelihood is given by

$$ L\left(\beta, \alpha \Big|D\right)={\displaystyle \prod_{i=1}^n{\displaystyle \prod_{j=1}^{m_i}{\left(\alpha {t}_{ij}^{\alpha -1} \exp \left({\eta}_{ij}\right)\right)}^{\delta_{ij}}}} \exp \left(- \exp \left({\eta}_{ij}\right){t}_{ij}^{\alpha}\right), $$

where *δ*_*ij*_ is the censoring indicator having a value 1 if the individual in the *j* − th cluster dies and 0 otherwise and *D* = (*t*, *X*, *δ*, *b*) denotes the complete data set with $t=\left({t}_{11},\dots, {t}_{n{m}_n}\right)\hbox{'}$, $\mathbf{X}=\left({X}_{11},\dots, {X}_{n{m}_n}\right)\hbox{'}$, $\delta =\left({\delta}_{11},\dots, {\delta}_{n{m}_n}\right)\hbox{'}$ and **b** = (*b*_1_, …, *b*_*n*_)’.

For the Uganda DHS data 2011, we assume that the time to death (*t*_*i*_) of children under the age of five follows a Weibull distribution. Given that *β* = (*β*_0_, *β*_1_, …, *β*_*n*_)^’^ is the vector of coefficients of the covariates considered for analysis, *β*_0_ is the intercept and *n* the number of covariates, we assume that all these coefficients have a normal prior with mean 0 and variance 0.001. We also assume a gamma prior with parameters 1 and 0.001 for the shape parameter *α* of the Weibull distribution *α*.

$$ {t}_i\sim Weibull\left(\alpha, {\lambda}_i\right), $$

Where *i* = 1, …, 6692.

## Appendix 3

### Confidence intervals for proportions

Given that *n* = 6692, the total number of children under-five in the dataset is large, the mortality rate of children under the age of five and its confidence interval was calculated as given below;

$$ \widehat{p}\pm z*\sqrt{\frac{\widehat{p}\left(1\hbox{-} \widehat{p}\right)}{n},} $$

where

$$ \widehat{p}=\frac{\mathrm{Total}\kern0.5em \mathrm{number}\kern0.5em \mathrm{o}\mathrm{f}\ \mathrm{deaths}\ \mathrm{f}\mathrm{o}\mathrm{r}\ \mathrm{children}\ \mathrm{under}\ \mathrm{the}\ \mathrm{age}\ \mathrm{o}\mathrm{f}\ \mathrm{f}\mathrm{ive}}{\mathrm{Toatal}\ \mathrm{number}\ \mathrm{o}\mathrm{f}\ \mathrm{children}\ \mathrm{under}\ \mathrm{age}\ \mathrm{o}\mathrm{f}\ \mathrm{f}\mathrm{ive}\ \mathrm{in}\ \mathrm{the}\ \mathrm{dataset}}. $$

The *z*-value is the 95 % standard normal distribution value level of confidence.
